# An RP-LC-UV-TWIMS-HRMS and Chemometric Approach to Differentiate between *Momordica* *balsamina* Chemotypes from Three Different Geographical Locations in Limpopo Province of South Africa

**DOI:** 10.3390/molecules26071896

**Published:** 2021-03-27

**Authors:** Pieter Venter, Kholofelo Malemela, Vusi Mbazima, Leseilane J. Mampuru, Christo J. F. Muller, Sylvia Riedel

**Affiliations:** 1 Biomedical Research and Innovation Platform, South African Medical Research Council, P.O. Box 19070, Tygerberg 7505, South Africa; malemela.kholofelo@mrc.ac.za (K.M.); Christo.Muller@mrc.ac.za (C.J.F.M.); 2 Department of Biochemistry, Microbiology and Biotechnology, University of Limpopo, Private Bag x1106, Sovenga 0727, South Africa; vusi.mbazima@ul.ac.za (V.M.); leseilane.mampuru@ul.ac.za (L.J.M.); 3 Division of Medical Physiology, Faculty of Medicine and Health Sciences, Stellenbosch University, P.O. Box 241, Cape Town 8000, South Africa

**Keywords:** *Momordica balsamina*, high-resolution mass spectrometry, reversed phases liquid chromatography, travelling wave ion mobility spectrometry, chemometric analysis, cytotoxicity assays

## Abstract

*Momordica balsamina* leaf extracts originating from three different geographical locations were analyzed using reversed-phase liquid chromatography (RP-LC) coupled to travelling wave ion mobility (TWIMS) and high-resolution mass spectrometry (HRMS) in conjunction with chemometric analysis to differentiate between potential chemotypes. Furthermore, the cytotoxicity of the three individual chemotypes was evaluated using HT-29 colon cancer cells. A total of 11 molecular species including three flavonol glycosides, five cucurbitane-type triterpenoid aglycones and three glycosidic cucurbitane-type triterpenoids were identified. The cucurbitane-type triterpenoid aglycones were detected in the positive ionization mode following dehydration [M + H − H_2_O]^+^ of the parent compound, whereas the cucurbitane-type triterpenoid glycosides were primarily identified following adduct formation with ammonia [M + NH_4_]^+^. The principle component analysis (PCA) loadings plot and a variable influence on projection (VIP) analysis revealed that the isomeric pair balsaminol E and/or karavilagen E was the key molecular species contributing to the distinction between geographical samples. Ultimately, based on statistical analysis, it is hypothesized that balsaminol E and/or karavilagen E are likely responsible for the cytotoxic effects in HT-29 cells.

## 1. Introduction

**Figure 1:**
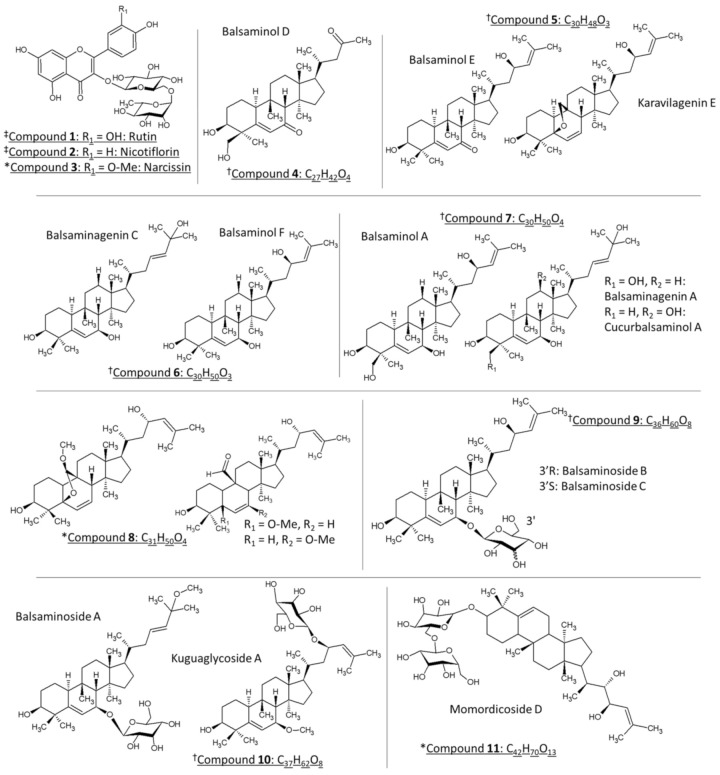
The chemical structures of the compounds identified in *M. balsamina* in the present work. Compounds marked with ‡ (**1** and **2**) have been tentatively identified in *M. balsamina* using LC-MS, whereas compounds marked with † have been previously isolated and characterized in *M. balsamina*. Lastly, compounds symbolized with * have been tentatively identified in the present work based on high and low collisional energy mass spectrometry and ultraviolet spectroscopy. Compounds **5**–**8** and **10** with similar molecular formulas are grouped together.

*Momordica balsamina*, commonly known as the balsam apple, is a perennial trailing herb that is native to tropical regions of Asia and widespread in southern Africa. Previous biological studies using the leaf extracts of *M. balsamina* revealed bioactive compounds with anti-hypertensive [1], anti-malaria [2,3,4,5,6], anti-cancer [7,8], anti-bacterial [9], schistosomicidal [10], and anti-diabetic properties [11]. Liquid chromatographic purification of the methanol leaf extract led to the isolation of the bioactive compounds which have been identified by nuclear magnetic resonance (NMR) and mass spectrometry (MS) as cucurbitane-type triterpenoids [1,2,12,13]. These molecular species were predominantly detected as sodium adducts using electrospray ionization (ESI). Structurally, these compounds are characterized by the tetracyclic cucurbitane skeleton, 19-(10→9β)-abeo-10α-lanost-5-ene, which varies in the number of hydroxy substituents and their glycosylated equivalents [13]. On the other hand, an LC-ESI-MS/MS analysis of the air-dried leaves, extracted with 80% methanol, revealed the presence of several polyphenols, including flavonoid glycosides and phenolic acids, such as chlorogenic acids, in *M. balsamina* [14,15]. Figure 1 represents the chemical structures that have been identified in the current work. Compounds **4**–**7**, **9** and **10** have been isolated and characterized previously in *M. balsamina* as cucurbitane-type triterpenoid aglycones (**4**–**7**) and their glycosidic equivalents (**9**, **10**) using nuclear magnetic resonance (NMR) and mass spectrometry (marked with † in Figure 1) [1,2,4,8,12]. The previously isolated triterpenoids showed significant antiproliferative and cytotoxic effects in a selection of cancer cells including gastric and pancreatic cancer cells as well as HT-29 colon cancer cells [7], while there is currently no literature regarding underlying mechanisms of action of these compounds.

The chemical complexity of natural products provides incentive to increase the separation power of one-dimensional liquid chromatography separations for increased metabolome coverage. One way to increase the separation power is by two-dimensional liquid chromatography, while on the other hand, one-dimensional liquid chromatography followed by ion mobility spectrometry can be a substitute [16]. Therefore, ion mobility spectrometry (IMS) has become an attractive tool, in combination with liquid chromatography for the non-targeted metabolome analysis of complex samples [17,18]. Ion mobility separates the gas phase ions according to size-to-charge ratio. More specifically, during ion mobility spectrometry a gas phase ion collides with an inert buffer gas while under the influence of a static electric field (drift tube ion mobility spectrometry, DTIMS) or a dynamic electric field (travelling wave ion mobility spectrometry, TWIMS), which results in a size-to-charge separation [19,20]. In this study a travelling wave ion mobility spectrometer (TWIMS) was used which works on the principle of a travelling pulse (wave) along a stacked ring ion guide that provides the ion propulsion compared to DTIMS which uses a static electric field. Combining IMS with LC-MS has the following benefits: (i) deconvolution of co-eluting chromatographic peaks to improve the confidence level of peak (feature) annotation [17,18,21], (ii) converting the arrival time into a collisional cross sections (CCS) value, which serves as another mode of identification [22] and (iii) increased duty cycle for low abundant compounds [20].

In this work, we employed an RP-LC-UV-TWIMS-HRMS and statistically based untargeted metabolomics approach to identify the molecular species and to differentiate between the chemical makeups of the three geographical chemotypes. This approach could help to identify possible chemotype markers for future biological activity, since it is well established that chemical composition can vary widely due to differences in local soil quality and other environmental conditions. In conjunction with this, we investigated the cytotoxicity of the three chemotypes in HT-29 colon cancer cells to identify possible chemotype markers with potential for future detailed investigation of biological activities. We also report for the first time the collisional cross sections (^TW^CCS_N2_) of the identified cucurbitane-type triterpenoids in *M. balsamina* using TWIMS.

## 2. Results and Discussion

### 2.1. Identification of Compounds

**Figure 2:**
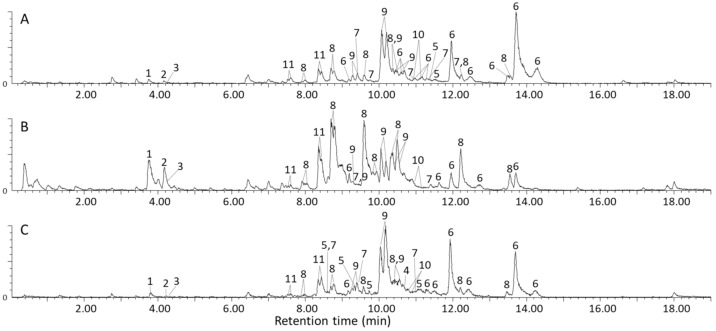
Base peak ion chromatograms obtained for the RP-LC analysis of *Momordica balsamina* from three different geographic areas in South Africa, named Letsitele (**A**), Goedplaas (**B**) and Mankweng (**C**). Peak labels correspond to Table 1, Table 2 and Table 3.

The base peak ion chromatograms obtained for the methanol extracts of Letsitele, Goedplaas and Mankweng chemotypes are presented in Figure 2A–C respectively, detected using RP-LC coupled to UV, TWIMS and HRMS. In total, 11 molecular species were identified, comprising three flavonol glycosides, five cucurbitane-type triterpenoid aglycones and three glycosides, all summarized in Table 1, Table 2 and Table 3. Furthermore, the 11 molecular species comprised a total of 43, 32 and 37 isomers for Letsitele, Goedplaas and Mankweng, respectively, obtained using a superficially porous RP-LC column. Identity confirmation of these molecular species was based on high-resolution MS data, CCS values and UV spectral information compared to previous literature reports [1,2,4,8,12,23,24,25,26,27,28,29,30,31]. For each identification, a confidence level is specified, which is based on criteria put forward by Schrimpe-Rutledge [32] and Schymanski et al. [33]. Briefly, level 1 is the unambiguous identification via the appropriate measurement of a reference standard; on level 2, an exact structure is proposed using orthogonal methods (UV, MS and IMS-MS); on level 3, the exact structure remains tentative; on level 4, an unambiguous molecular formula can be assigned and finally, level 5 states that no unequivocal information about the structure or formula exists.

Compounds **1**, **2** and **3**, were putatively identified (level 2) as quercetin 3-*O*-rutinoside (*m*/*z* 611.1604, 0.4 ppm), kaempferol 3-*O* rutinoside (*m*/*z* 595.1671, 1.3 ppm) and isorhamnetin 3-*O* rutinoside (625.1782, 2.1 ppm), respectively, based on MS [27,28,29,30] and UV data [23,24] shown in Figures S1 and S2 from Supplementary Material.

Further supporting the presence of quercetin 3-*O*-rutinoside (**1**) in *M. balsamina* is the CCS value of 230.1 Å (Table 1), which compares well with previously reported values of 230.3, 230.8 and 231.0 Å, which also used poly-DL-alanine as calibrant [25,26,34]. However, drift tube IMS (DTIMS) is the benchmark IMS technique that does not require a calibrant to calculate the CCS value. Instead, the CCS value can be measured directly from the drift time to avoid calibration errors. Using DTIMS, a CCS value of 236.2 Å was obtained for quercetin 3-*O*-rutinoside, which indicates a percentage difference of 2.6% when compared to our experimental CCS value [35]. This might therefore suggest that poly-DL-alanine is not the ideal calibrant for calculating the accurate CCS values of flavonoid *O*-diglycosides.

All the cucurbitane-type triterpenoids previously isolated from *M. balsamina* were detected as sodium adducts [M + Na]^+^ [1,2,4,8,12]. However, our mass spectral results revealed the production of several ionic species, which includes the dehydration [M + H − nH_2_O]^+^ of cucurbitane-type triterpenoids (**4**–**7**) and the formation of ammonia [M + NH_4_]^+^, sodium [M + Na]^+^ and potassium [M + K]^+^ adducts in the case of cucurbitane-type triterpenoid glycosides (**9**–**11**) (Table 1, Table 2 and Table 3). Although these compounds have been unambiguously characterized in *M. balsamina* using NMR spectroscopy, the lack of reference spectra describing the mass spectrometric fragmentation afforded these compounds only a tentative identification (level 3). Furthermore, numerous retention times were recorded for each isomeric species (compounds **5**–**11**). Therefore, for complete structural elucidation, NMR is crucial to determine the type isomer but requires time-consuming purification procedures which falls outside the scope of this study.

Evident from the base peak ion chromatograms (Figure 2) is the extensive co-elution of the cucurbitane-type triterpenoid aglycones (compounds **5**–**8**) and their related glycosides (compounds **9**–**11**), which will be discussed in more detail in the following paragraphs. Nevertheless, from the two-dimensional RP-LC × TWIMS plot (Figure S3) it is evident that sufficient size-to-charge separation was achieved in the second dimension for the aglycones and their glycosidic counterparts. This approach significantly simplified data interpretation by allowing the acquisition of clean mass spectra by filtering the MS data according to arrival time.

**Figure 3:**
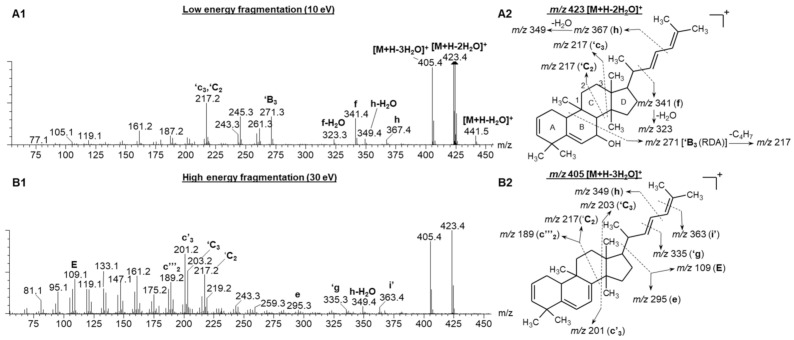
The fragmentation spectra of compound **6** under low (**A1**) and high (**B1**) energy CID conditions. (**A2**) represents the proposed fragmentation pattern of *m*/*z* 423 [M + H − 2H_2_O]^+^ under low energy conditions and (**B2**) illustrates the fragmentation pattern of *m*/*z* 405 [M + H − 3H_2_O]^+^ under high energy conditions.

Figure 3(A1,B1) represents the low and high collision energy mass spectra of compound **6**, tentatively identified as balsaminol F (cucurbita-5,25(*E*)-diene-3β,7β,23-triol) and balsaminagenin C (cucurbita-5,23(*E*)-diene-3β,7β,25-triol) [1,4]. Evident from the low and high energy mass spectra (Figure 3(A1)) is the consecutive loss of three water molecules producing fragment ions at *m*/*z* 441.5 [M + H − H_2_O]^+^, *m*/*z* 423.4 [M + H − 2H_2_O]^+^, and *m*/*z* 405.4 [M + H − 3H_2_O]^+^ confirming the presence of three hydroxyl groups. Similarly, the high and low energy spectra of compound **7** (Figure S4), previously identified as balsaminagenin A, cucurbalsaminol A and balsaminol A produced fragment ions at *m*/*z* 457.5 [M + H − H_2_O]^+^, *m*/*z* 439.4 [M + H − 2H_2_O]^+^, *m*/*z* 423.4 [M + H − 3H_2_O]^+^, and *m*/*z* 405.4 [M + H − 4H_2_O]^+^ which reveals the presence of four hydroxyl groups. For compounds **4** and **5,** the number of hydroxyl groups could not be determined due to co-elution and similar arrival times, which precluded the acquisition of unambiguous mass spectra. It is well known that this fragmentation behavior is prominent for triterpenoid derivatives where the base peak was characterized by a prominent dehydrated ([M + H − nH_2_O]^+^) *m*/*z* fragment [36,37,38,39,40,41,42].

Aside from the dehydration reactions, Griffiths’ charge remote fragmentation (CRF) nomenclature used for cholesterol characterization was adopted in the current study to hypothesize the C-C bond cleavages of cucurbitane-type triterpenoids [36]. Figure 3 illustrates the CRF fragmentation behavior of compound **6** under low and high collision energy.

For the sake of simplicity, only the dominant fragment ion will be briefly discussed in the following paragraph. Under low energy conditions, the fragment ion at *m*/*z* 271.2 (‘B_3_), could be formed due to the B ring undergoing a retro-Diels Alder reaction. Subsequently, a neutral loss of C_4_H_7_ from the aliphatic side chain produced the *m*/*z* 217.2 peak under both low and high energy conditions (Figure 3(A1,B1)). Another possible scenario to produce *m*/*z* peak at 217.2 is the cleavage of ring C, at positions ′C_2_ and ′c_3_. The predominant fragment ions at *m*/*z* 201 and 203 formed under high energy collision induced dissociation (CID) could be due to the cleavage of C8-C14 (c′_3_) and C12-C13 (′C_3_) bonds (Refer to Figure S5 for the carbon numbering system). Further fragmentation of *m*/*z* 201 and 203 resulted in *m*/*z* peaks 14 Da apart, which corresponds to the neutral loss of C_n_H_2n_ units. Furthermore, the low energy spectrum (Figure 3(A1)) of compound 6 showed the fragmentation of the single bonds in the aliphatic side chain, producing fragment ions at *m*/*z* 367.4 (h) and *m*/*z* 341.4 (f). These fragment ions are similar for all the detected cucurbitane-type triterpenoids (**5**–**8**), only differing in the fragment ion intensities.

Another abundant species, compound **8**, showed an HRMS peak at *m*/*z* 509.3618 (3.3 ppm) corresponding to a molecular formula of C_31_H_50_O_4_Na. Zhang et al. [43] and Jiang et al. [44] have isolated and characterized three curcurbitane aglycone isomers from the aerial parts of *Momordica charantia* L. with the same molecular formula of C_31_H_50_O_4_. They were characterized using HRMS and NMR spectroscopy as (19*R* or *S*)-5b,19-epoxy-19-methoxycucurbita-6,24-dien-3b,23-diol, 3β,23-dihydroxy-5-methoxycucurbita-6,24-dien-19-al and 3β,23-dihydroxy-7-methoxycucurbita-5,24-dien-19-al (Figure 1) which produces solely sodiated pseudo-molecular ions [M + Na]^+^ using ESI. The fragmentation spectrum of compound **8** illustrated in Figure S6 displays the same fragment ions compared to compound **6** illustrated in Figure 3(B1). Therefore, considering that this compound was also isolated from the aerial parts and revealed similar fragment ions compared to the identified cucurbitanes, compound **8** was reasonably deduced to be a curcurbitane-type triterpenoid with a molecular formula of C_31_H_50_O_4_ (level 3).

As already mentioned, several glycosylated curcurbitanes (**9**, **10, 11**), namely balsaminosides A, B, C, kuguaglycoside A and momordicoside D were tentatively identified based on their adduct formation with NH_4_^+^, Na^+^ and K^+^. The proposed nomenclature by Domon and Costello will be used to illustrate the fragmentation of the cucurbitane-type triterpenoids glycosides [45]. The low and high energy spectra for compound **9** (Figure S7A,B) revealed the presence of the aglycone unit at *m*/*z* 457 [**Y_0_**]^+^, whereas the high energy spectrum (Figure S7B) revealed the presence of the carbohydrate fragment at *m*/*z* 179 [C1]^+^. Regarding compound **10**, no clean filtered mass spectrum could be obtained. Finally, we tentatively assigned compound **11** as a cucurbitane diglycoside based on the calculated molecular formula of C_42_H_70_O_13_Na^+^ (*m*/*z* 805.5133, 1.8 ppm), which is similar to momordicoside D that was isolated and characterized in the seeds of *Momordica charantia* L. using NMR and HRMS [31]. The low energy spectrum revealed the loss of one sugar moiety producing a fragment ion at *m*/*z* 603.4 [Z_1_] (Figure S8A), whereas, the carbohydrate unit at *m*/*z* 179 [C_1_] was observed under high collision energy (Figure S8B).

Summarized in Table S1 are the ^TW^CCS_N2_ values derived for flavonoid *O*-glycosides, cucurbitanes-type triterpenoids and their glycosidic counterparts using poly-DL-alanine as calibrant. However, the different physical properties between poly-DL-alanine and the compounds identified in this study could lead to errors in calibrated CCS values [22,46]. Nevertheless, another beneficial feature of incorporating ion mobility into the LC-MS workflow, apart from filtered spectra, becomes apparent when examining compounds **7** and **11**. Figures S9(A1,B1) show the two-dimensional separation space obtained by RP-LC × TWIMS for compounds **7** and **11**, respectively, as well the corresponding extracted ion arrival time plots, Figure 3(A2,B2). This indicates that ion mobility was able to differentiate between the isomers of compounds **7** and **11**, based on multiple arrival times due to structural effects like size and shape [47].

### 2.2. Statistical Analysis for Identification of Chemotype Markers

Visual inspection of the mass spectra shown in Figure S10 for Letsitele (**A**), Goedplaas (**B**), and Mankweng (**C**) shows that the most abundant species in the methanol extract of *M. balsamina* are compounds **6** and **8**, identified as balsaminagenin C (**6**) and/or balsaminol F (**6**) as well as a newly identified compound **8**. Another visual difference between these samples is the relatively high abundance of rutin (**1**, *m*/*z* 611) and compound **8** (*m*/*z* 509) in Goedplaas relative to the other two chemotypes. Other prominent species present in all three chemotypes are the cucurbitane aglycones detected at *m*/*z* 439 (**5**), 457 (**7**) and the cucurbitane diglycosides at *m*/*z* 800 (**11**).

**Figure 4:**
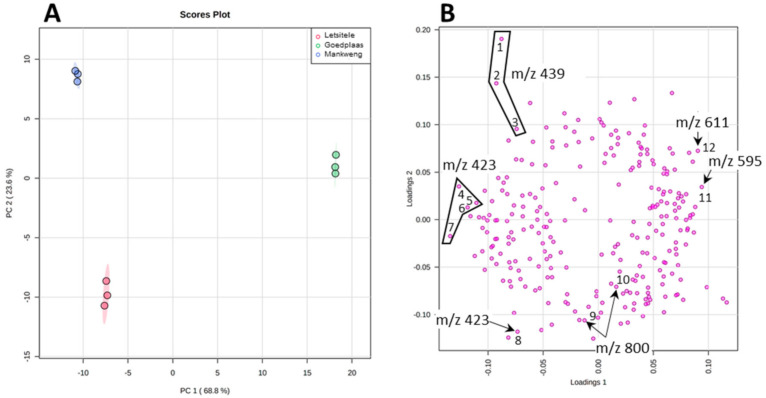
The PCA scores (**A**) and loading (**B**) plot of the three chemotypes. The numbers refer to the boxplots of each specific features illustrated in Figure S11.

In order to statistically differentiate between these geographical samples, we applied XCMS for preliminary data processing involving future detection, retention time correction and peak alignment [48,49,50]. Using the XCMS parameters specified section in Section 3.5 (data processing and statistical analysis), 823 features were identified and exported as an excel spreadsheet. A manual inspection of the exported data set was done using the knowledge that was gained during the MS characterization of the compounds, in particular, the adduct and product ion formation. Therefore, a single feature manifesting as multiple features due to adduct or product ion formation as well as isotopic peaks were identified and edited so that each compound is represented by a single feature. Following this amendment, a total of 286 features were left and uploaded into MetaboAnalyst for statistical analysis [51]. PCA score plot displayed in Figure 4A revealed a clear separation between the three chemotypes: Letsitele, Goedplaas and Mankweng. The two principal components of the PCA score plot, PC1 (68.8%) and PC2 (23.6%) represented a substantial 92.4% of the variance. Examining the PCA loadings plot (Figure 4B) revealed that some of the biggest contributors to PC1 are the flavonoid glycosides, rutin (**1**) and nicotiferin (**2**) and compound **6** (*m*/*z* 423). PC1 also explains the largest variance between Goedplaas on the one hand and Letsitele and Mankweng chemotypes on the other hand.

PC2 explained best the variation between all three chemotypes, where its respective loading plot revealed the molecular species contributing to PC2 as compound **5** (*m*/*z* 439). It is evident from the boxplots illustrated in Figure S11 that compound **5** is relatively more abundant in Mankweng compared to both Letsitele and Goedplaas chemotypes. Furthermore, the variable of importance (VIP) approach revealed that compound **5** (*m*/*z* 439), at retention times of 11.2 and 9.33 min, possessed the highest VIP scores of 3 and 2.5, respectively, as well as another isomeric species of compound **5** at a retention time of 8.55 with a VIP score of 1.7 (highlighted in blue, Figure S12). Other identified compounds that also contribute to potential differences between the three chemotypes are compounds **6** (*m*/*z* 423) eluting at 8.44 and 16.63 min with a VIP score of 1.7 and 1.6, respectively (Figure S12). Finally, compound **11** (*m*/*z* 800) identified as a cucurbitane diglycoside revealed a VIP score of 1.6.

#### Cytotoxicity Study

**Figure 5:**
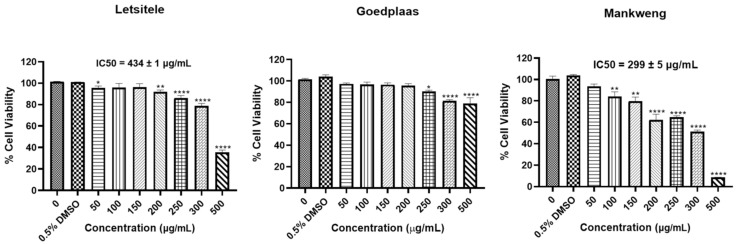
Effect of the three chemotypes of *M. balsamina* on viability of HT-29 colon cancer cells. Cells were treated with extract concentrations between 50 and 500 µg/mL and 0.5% DMSO as the vehicle control for 24 h. Cell viability was determined using the MTT assay. Each data point represents the mean ± standard deviation (S.D) of three independent experiments, performed in triplicate. * *p* ≤ 0.05, ** *p* ≤ 0.01 and **** *p* ≤ 0.0001 indicate significant differences to the dimethyl sulfoxide (DMSO) control.

The cytotoxic effects of the *M. balsamina* extracts were assessed using the MTT assay (Figure 5). The results showed a statistically significant reduction in cell viability of HT-29 cells treated with the *M. balsamina* methanol extract from Goedplaas only from concentrations of 250 μg/mL and above. Treatment with *M. balsamina* methanol extract from Letsitele reduced HT-29 cell viability at 50 μg/mL (* *p* ≤ 0.05), 200 μg/mL (** *p* ≤ 0.01) and from 250 μg/mL and above (**** *p* ≤ 0.0001) with an IC_50_ of 434 ± 1 μg/mL. Treatment with *M. balsamina* methanol extract from Mankweng resulted in a significant reduction in HT-29 cell viability at concentrations from 100 μg/mL (** *p* ≤ 0.01) and above with an IC_50_ of 299 ± 5 μg/mL. The *M. balsamina* methanol extract from Mankweng displayed the highest cytotoxicity in HT-29 cancer cells when compared to the methanol extracts from Letsitele and Goedplaas.

Taking into consideration that compound **5** is relatively more abundant in the extract originating from Mankweng compared to the other two geographical samples and has the highest VIP scores, it was hypothesized that this compound may be responsible for the toxicity in HT-29 cells. Both compounds **5** and **6** previously showed significant cytotoxic effects in MCF-7 breast cancer [2] and L5178Y mouse T-lymphoma cells [1], respectively, while our study showed relatively low overall toxicity of the extracts in HT-29 colon cancer cells.

## 3. Material and Methods

### 3.1. Material and Reagents

HPLC-grade acetonitrile and methanol were purchased from Romil (Microsep, Johannesburg, Gauteng, South Africa). Formic acid was obtained from Merck (Darmstadt, Hessen, Germany) and deionised water was obtained using a Milli-Q water purification system (Millipore, Milford, MA, USA). Poly-DL-alanine used for ^TW^CCS_N2_ calibration was purchased from Sigma-Aldrich (St. Louis, MO, USA). *M. balsamina* leaves were collected from Letsitele (23°57′51″ S 30°22′39″ E), Goedplaas (23°31′34.0″ S 30°03′20.2″ E) and Mankweng (23°53′47.7″ S 29°43′44.1″ E) in the Limpopo province of South Africa and subsequently identified at the Larry Leach Herbarium at the University of Limpopo.

### 3.2. Sample Extraction

Ten grams of each *M. balsamina* species were separately extracted with 70 mL methanol in a Soxhlet apparatus for 5 h. Afterwards, most of the methanol was evaporated in a Rotavapor until the volume was less than 15 mL. The final volume was adjusted to 15 mL using methanol and stored at −80 °C until analysis. The samples were diluted to 50% water and filtrated through 0.22 μm low binding PES membranes prior to LC-MS analysis.

### 3.3. RP-LC-TWIMS-HRMS Conditions

RP-LC-TWIMS-HRMS analyses were performed on an Acquity UPLC system hyphenated to a photodiode array (PDA) detector (500 nL flow cell, 10 mm path length) and a Synapt G2 quadrupole time-of- flight (Q-TOF) mass spectrometer via an ESI source (Waters, Milford, MA, USA). Separations were performed on a Kinetex (50 mm × 2.1 mm i.d., 1.7 μm·dp) superficially porous column (Phenomenex, Torrance, CA, USA) using mobile phase A which consists of 0.1% formic acid in water and mobile phase B consisting of acetonitrile. The following linear gradient was used: 1–95% B (0–20 min), 95% B (25 min), 95–1% B (25.01–30 min) at a flow rate of 0.4 mL/min with the total flow directed to the MS source. UV detection was performed from 230 to 500 nm at a 20 Hz acquisition rate. Nitrogen at a temperature of 275 °C was used as desolvation with a flow rate of 650 L/h. MS data were acquired using positive ionization with a cone voltage and capillary voltage of 15 V and 3 kV, respectively. A scan time of 0.2 s was used to acquire data from 200–2000 amu. For high energy spectra (MS^E^), an energy ramp from 10–30 eV was used. Leucine enkephalin was used as the lock mass calibrant. The instrument was operated at a resolving power of 18,000 (*m*/*z* 554).

Nitrogen at a flow rate of 90 mL/min was used as the buffer gas for ion mobility separations with a mobility T-Wave velocity and wave height of 448 m/s and 37.1 V, respectively. Helium at a flow rate of 180 mL/min was used to maintain the pressure in the helium cell.

### 3.4. Collisional Cross-Section Determination

^TW^CCS_N2_ calibration was performed using Poly-DL-alanine at a concentration of 10 mg/L prepared in deionized water. A protocol developed by Ruotolo et al. [52] was used to calculate the CCS values. Very briefly, an exponential factor (X) was determined by plotting ln t’_d_ vs. ln Ω’, which in turn was used to calculate the corrected arrival time (t’’_d_). The corrected arrival time (t’’_d_) was plotted against the known Ω values of Poly-DL-alanine.

### 3.5. Data Processing and Statistical Analysis

Data acquisition and processing were performed using MassLynx (v. 4.1) and DriftScope (v. 2.1) software (Waters Corp., Milford, MA, USA). Low and high collision energy mass spectra were filtered as a function of arrival time using Driftscope (v. 2.1) to obtain ‘clean’ mass spectra. For statistical analysis, the MSConvert tool from the freely available Proteowizard software was used to convert the vendor format (.raw) to the cross- platform mzML format [53]. Once converted, XCMS was used to perform future detection, retention time correction and peak alignment [48,49,50]. Briefly, for high-resolution MS feature detection, the CentWave algorithm was used, the maximum allowed *m*/*z* deviation was 5 ppm, and the minimum and maximum chromatographic peak widths were set to 2 and 15 s, respectively, with a prefilter intensity of 500. Retention time correction was performed using the obiwarp algorithm. For peak alignment, the width of overlapping *m*/*z* slices was set to 0.015 and the allowable retention time deviation was set to 5 s and a minfrac setting of 0.5. Once the data were processed in XCMS, it was exported and uploaded to the MetaboAnalyst software package for peak normalization and statistical analysis [51]. During peak normalization, the dataset was median-centered, log-transformed and pareto scaled.

Principal component analysis (PCA) was performed to reveal discriminate biomarkers between the three chemotypes. To determine what compound possessed the highest discriminatory effect between the three chemotypes, a variable importance in projection (VIP) analysis was performed.

## 4. Toxicity Study

### 4.1. Cell Culture and Maintenance and Treatment

Human colon HT-29 colon cancer cells (ATCC^®^ HTB-38™) were grown in 75 cm^3^ flasks containing Dulbecco’s Modified Eagle’s Medium (DMEM), supplemented with 10% (*v*/*v*) heat-inactivated fetal bovine serum (FBS) and maintained in a tissue culture incubator at 37 °C in humidified air containing 5% CO_2_. The cells were sub-cultured every fourth day at a ratio of 1:8 and refreshed every second day. The cells were used from passage 150 to P158 to avoid phenotypic drift caused by repeated sub-culturing of the cells. Cells were seeded in 96-well plates in a volume of 100 µL of cells per well (1 × 10^5^ cells per ml) and allowed to attach for 72 h, whereafter they cells were treated with 50–500 µg/mL of the respective *M. balsamina* extracts for 24 h. The dried extracts were dissolved in 100% DMSO (100 mg/mL) which was then aliquoted and kept at −20 °C until use. The stock solutions were subsequently diluted to the respective concentration in media with a final DMSO concentration of 0.5%.

### 4.2. Cytotoxicity Assay

The effect of the *M. balsamina* extracts on the viability of HT-29 cells was evaluated by measurement of the metabolic activity using the 3-(4,5-dimethylthiazol-2-yl)-2,5-diphenyltetrazolium (MTT) assay as described by Mosmann [54], with slight modifications. Following the 24 h treatment, the cell culture media was removed and 100 µL of a 1 mg/mL of MTT solution in 1x Dulbecco’s Phosphate-Buffered Saline (DPBS) added and incubated for 30 min in a tissue culture incubator. The MTT solution was then removed and 200 µL of dimethyl sulfoxide (DMSO) added to dissolve the formazan crystals. For quantification, the absorbance was measured at 570 nm using a SpectraMax i3x pate reader (Molecular Devices, LLC, Sunnyvale, CA, USA). Data were expressed as percentage viability calculated using the formula [54]:

$$\%Viability=\;\frac{Absorbance\;of\;treated\;cells}{Absorbance\;of\;untreated\;cells}\;\times 100$$

### 4.3. Statistical Analysis

Three independent experiments were performed in triplicate and expressed as mean ± S.D. Statistically significant differences between untreated controls and treatments were determined using GraphPad prism version 8.4.3. (GraphPad Software, San Diego, CA, USA) software by one-way ANOVA. Differences between mean of untreated and treated cells were considered significant at *p* ≤ 0.05 (*), *p* ≤ 0.01 (**) and *p* ≤ 0.001 (***) and *p* ≤ 0.0001 (****). IC_50_ values were calculated by plotting activity against log concentration of the compounds in a line graph using GraphPad Prism using Non-linear regression (four parameter curve fit) with dose response-inhibition selected and variable slope.

## 5. Conclusions

*M. balsamina* is widely used as a vegetable and as part of traditional medicines in Southern Africa [55], which has sparked interest in the scientific elucidation of its bioactive compounds for future bioprospecting studies [1,2,3,4,5,6,7,8,9,10,11]. The RP-LC-UV-TWIMS-HRMS workflow was successfully employed for the characterization of compounds in methanol extracts of three *M. balsamina* chemotypes. These compounds included cucurbitanes-type triterpenoid aglycones and glycosides as well as flavanol glycosides. TWIMS was also effectively employed to determine the ^TW^CCS_N2_ values of the cucurbitane aglycones and glycosides for the first time in *M. balsamina*. Apart from using CCS values as an additional characterization tool, the advantage of employing ion mobility spectrometry following UHPLC separation is the obtainment of clean mass spectra by filtering the MS data according to arrival time. This significantly improves the data quality and interpretation, thereby limiting the potential of false positive feature detection using multivariate statistical analysis. Furthermore, by combining multivariate statistical analysis with the toxicity study we were able to identify possible cytotoxic compounds of interest. Future outcomes include confirming whether compound 5 elicits cytotoxic effects in HT-29 cells and whether antagonistic effects of other compounds present in the extracts could affect cytotoxicity. Finally, due to structural differences between the identified compounds and Poly-DL-alanine, it is imperative to determine the validity of this approach by using a DTIMS instrument in future.

## Figures and Tables

**Table 1:** Flavonol Glycosides Identified in *M. balsamina* using RP-LC-TWIMS-HRMS.

Compound Name (No)	Molecular Formula	^12^C Mass	Retention Time (min)	Exp. [M + H]^+^	Mass Error (ppm)
Quercetin 3-*O*-rutinoside (Rutin) (**1**)	C_27_H_30_O_16_	610.1534	3.76 ^L,G,M^	611.1604	−0.4
Kaempferol 3-*O*-rutinoside (Nicotiflorin) (**2**)	C_27_H_31_O_15_	594.1585	4.17 ^L,G,M^	595.1671	1.3
Isorhamnetin 3-*O*-rutinoside (**3**)	C_28_H_33_O_16_	624.1690	4.29 ^L,G,M^	625.1782	2.1

Retention times in Letsitele ^L^, Goedplaas ^G^, Mankweng ^M.^

**Table 2:** Cucurbitane-type Triterpenoid Aglycones Identified in *M. balsamina* using RP-LC-TWIMS-HRMS.

Compound No. (Name)	Molecular Formula	^12^C Mass	Retention Time (min)	Exp. [M + H − H_2_O]^+^	Mass Error (ppm)	Exp. [M + Na]^+^	Mass Error (ppm)
Compound **4** (Balsaminol D)	C_27_H_42_O_4_	430.3083	10.69^M^	413.3010	−4.1		
Compound **5** (Balsaminol E, Karavilagenin E)	C_30_H_48_O_3_	456.3603	(11.20, 11.55, 14.79) ^L^, (8.01, 10.25) ^G^, (8.55, 9.33, 9.74, 11.21) ^M^	439.3555	−3.5		
Compound **6** (Balsaminagenin C, Balsaminol F)	C_30_H_50_O_3_	458.3760	(9.18, 10.37, 10.60, 10.68, 10.95, 11.17, 11.29, 11.96, 12.46, 13.51, 13.71, 14.29) ^L^, (9.18, 11.60, 11.96, 12.75, 13.71) ^G^, (9.18, 11.29, 11.50, 11.96, 12.44, 13.71, 14.29) ^M^	441.3719 *	−1.8		
Compound **7** (Balsaminagenin A, Cucurbalsaminol A, Balsaminol A)	C_30_H_50_O_4_	474.3709	(9.44, 9.83, 11.00, 11.35, 12.26) ^L^, (9.44, 11.35) ^G^, (8.56, 9.44, 11.00, 11.35) ^M^	457.3670	−1.3		
Compound **8**	C_31_H_50_O_4_	486.3709	(7.89, 7.97, 8.69, 8.77, 9.59, 10.37, 10.49, 12.23, 13.56) ^L^, (7.89, 7.97, 8.77, 9.59, 9.83, 9.93, 10.37, 10.49, 12.21, 13.54) ^G^, (7.89, 7.98, 8.69, 8.77, 9.57, 10.33, 10.46, 12.20, 13.53) ^M^			509.3618	3.3

Retention times in Letsitele ^L^, Goedplaas ^G^, Mankweng ^M^, ^*^ Predominate ionic species detected for compound **6** is *m*/*z* 423 [M + H − 2H_2_O]^+^.

**Table 3:** Cucurbitane-type Triterpenoids Glycosides Identified in *M. balsamina* using RP-LC-IMS-HRMS.

Compound No. (Name)	Molecular Formula	^12^C Mass	Retention Time (min)	Exp. [M + NH_4_]^+^	Mass Error (ppm)	Exp. [M + Na]^+^	ppm	Exp. [M + K]^+^	Mass Error (ppm)
Compound **9** (Balsaminoside B and C)	C_36_H_60_O_8_	620.4288	(9.29, 9.41, 10.07, 10.19, 10.43, 10.60) ^L^, (9.28, 9.40, 10.06, 10.18, 10.42, 10.59) ^G^, (9.28, 9.39, 10.02, 10.18, 10.38, 10.55) ^M^	638.4632	0.0	643.4183	0.4	659.3943	3.5
Compound **10** (Balsaminoside A and Kuguaglycoside A)	C_37_H_62_O_8_	634.4445	(10.96, 11.17) ^L^, (10.97, 11.16) ^G^, (10.93, 11.16) ^M^	652.4774	−2.1	657.4243	−14.3	673.4089	3.9
Compound **11** (Momordicoside D)	C_42_H_70_O_13_	782.4816	(7.53, 7.59, 8.35, 8.44) ^L^, (7.54, 7.59, 8.35, 8.46) ^G^, (7.53, 7.59, 8.35, 8.43) ^M^	800.5133	−2.7	805.4694	−1.8	821.4448	0.0

Retention times in Letsitele ^L^, Goedplaas ^G^, Mankweng ^M^.

## Data Availability

Data available on request.

## Supplementary Materials

The following are available online, **Figures S1**–**S12** and **Table S1**: **Figure S1**: Low-energy CID (4 eV) spectra of quercetin 3-*O*-rutinoside (**A**), kaempferol 3-*O*-rutinoside (**B**), and isorhamnetin 3-*O*-rutinoside (**C**). ^#^Indicates fragment ions from compound **2**, **Figure S2**: UV spectra of Quercetin 3-*O*-rutinoside (A), Kaempferol 3-*O*-rutinoside (B), and Isorhamnetin 3-*O*-rutinoside (C) at retention times of 3.76, 4.17 and 4.29 min, respectively, **Figure S3**: The two-dimensional RP-LC × TWIMS contour plot of Letsitele, Goedplaas and Mankweng, **Figure S4**: MSE spectrum of compound 7 which displays the loss of 4 water molecules, **Figure S5**: Illustrating the carbon numbering system for the cucurbitane skeleton, **Figure S6**: Fragmentation spectrum of compound 8, **Figure S7**: The low (A) and high (B) collision energy MS spectra of compound 9, identified as a monoglycosylated cucurbitanes-type terpenoids. C represents the C-C bond cleavages, **Figure S8**: The low (i) and high (ii) energy spectra obtained for cucurbitane diglycosides and the proposed fragmentation pattern (iii), **Table S1**: The calculated TWCCSN2 values using Poly-DL-alanine as calibrant for compounds 1–11, **Figure S9**: Extracted ion RP-LC × ion mobility contour plots for a curcurbitane aglycone (A1) and curcurbitane glycoside (B1). Panel A2 and B2 show the corresponding extracted ion arrival time plot, **Figure S10**: The QTOF MS spectra of Letsitele (A), Goedplaas (B) and Mankweng (C) showing the dominant ionic species in Momordica balsamina in the positive ionization mode, **Figure S11**: Boxplots illustrating the normalized concentration for selected/identified species in M. balsamina, **Figure S12**: Variable importance in projection (VIP) plot.
