# Supplementary material for: An RP-LC-UV-TWIMS-HRMS and Chemometric Approach to Differentiate between Momordica balsamina Chemotypes from Three Different Geographical Locations in Limpopo Province of South Africa
Source: Molecules. 2021 Mar 27;26(7):1896. doi: 10.3390/molecules26071896 (PMC8036689; doi:10.3390/molecules26071896)
Supplement: Supplementary file 1 [file molecules-26-01896-s001.pdf]

molecules

Supplementary Materials

An RP-LC-UV-TWIMS-HRMS and Chemometric Approach to Differentiate between *Momordica balsamina* Chemotypes from Three Different Geographical Locations in Limpopo Province of South Africa

Pieter Venter [1,*], Kholofelo Malemela [1,2], Vusi Mbazima [2], Leseilane J. Mampuru [2], Christo J.F. Muller [1,3] and Sylvia Riedel [1,3,*]

[1] Biomedical Research and Innovation Platform, South African Medical Research Council, P.O. Box 19070, Tygerberg 7505, South Africa; malemela.kholofelo@mrc.ac.za
[2] University of Limpopo, Department of Biochemistry, Microbiology and Biotechnology, Private Bag x1106, Sovenga, 0727, South Africa; vusi.mbazima@ul.ac.za (V.M.); leseilane.mampuru@ul.ac.za (L.J.M.)
[3] Division of Medical Physiology, Faculty of Medicine and Health Sciences, Stellenbosch University, PO Box 241, Cape Town 8000, South Africa; Christo.Muller@mrc.ac.za
* Correspondence: pieter.venter@mrc.ac.za(P.V); Sylvia.Riedel@mrc.ac.za(S.R.); Tel +27 21 938 0421(P.V.); +27 21 938 0844(S.R.)

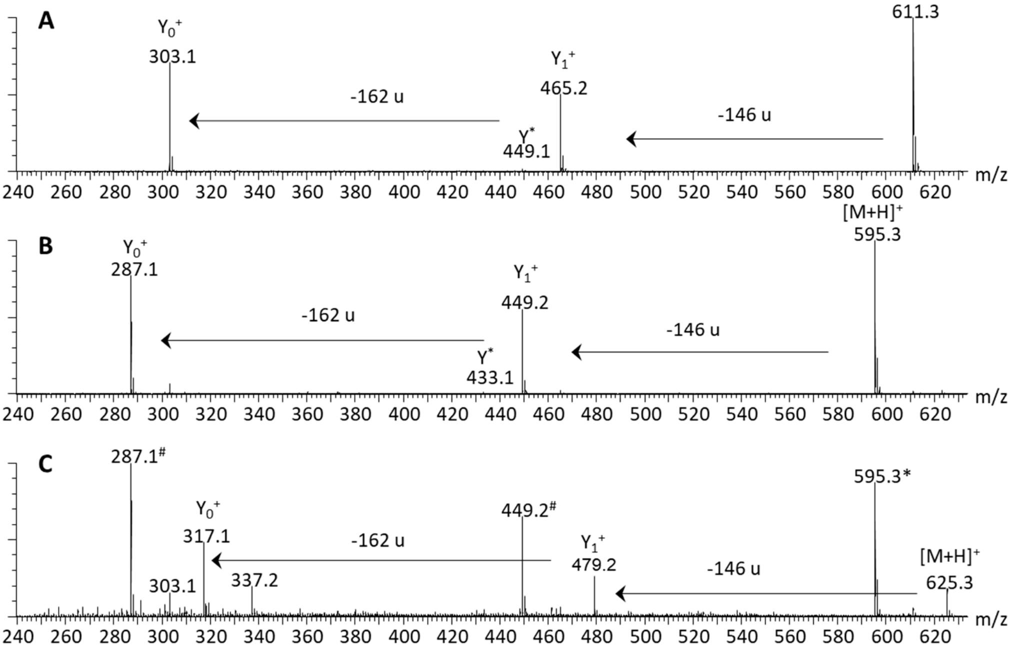

Figure S1. Low-energy CID (4 eV) spectra of quercetin 3-O-rutinoside (**A**), kaempferol 3-O-rutinoside (**B**), and isorhamnetin 3-O-rutinoside (**C**). #Indicates fragment ions from compound **2**.

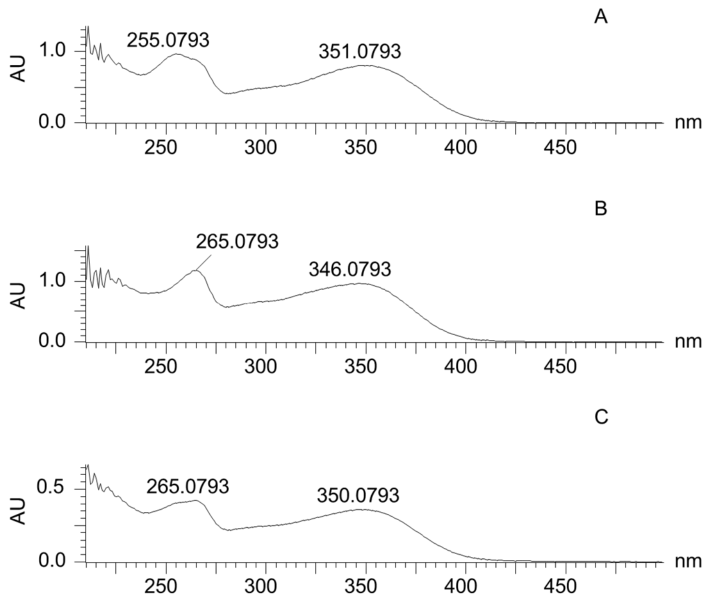

Figure S2. UV spectra of Quercetin 3-O-rutinoside (**A**), Kaempferol 3-O-rutinoside (**B**), and Isorhamnetin 3-O-rutinoside (**C**) at retention times of 3.76, 4.17 and 4.29 min, respectively.

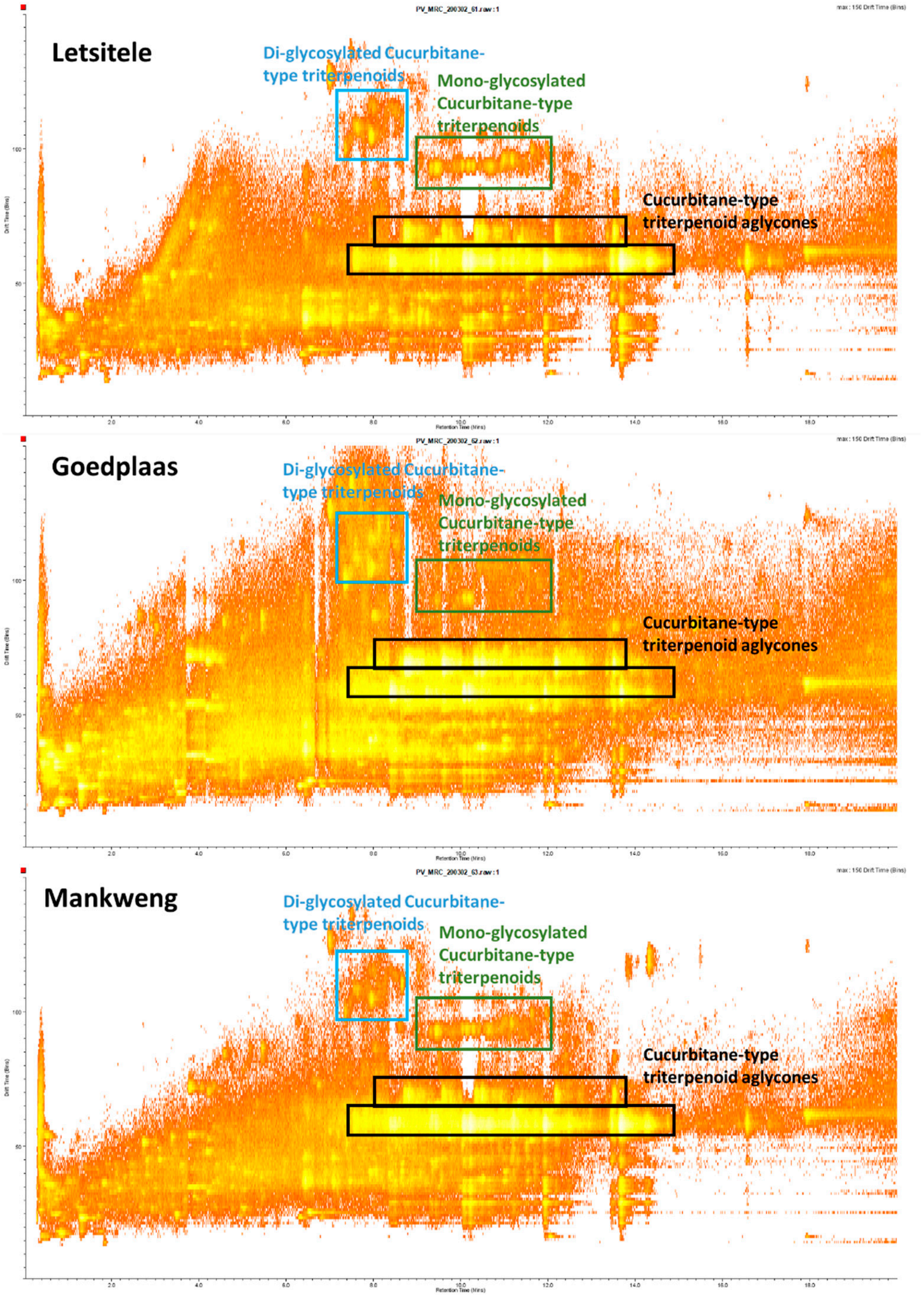

Figure S3. The two-dimensional RP-LC × TWIMS contour plot of Letsitele, Goedplaas and mankweng.

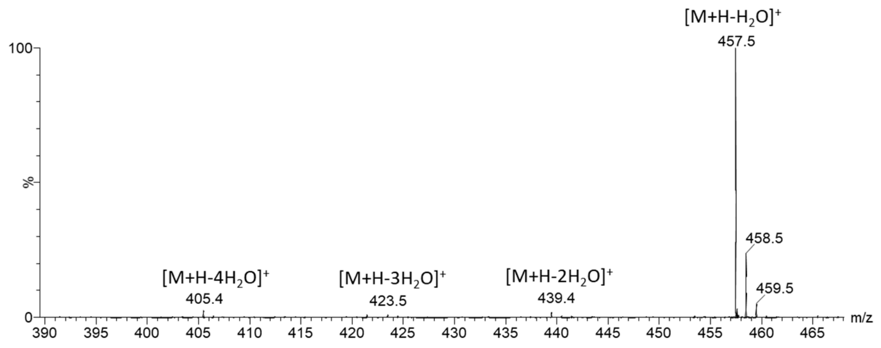

Figure S4. MS^E spectrum of compound **7** which displays the loss of 4 water molecules.

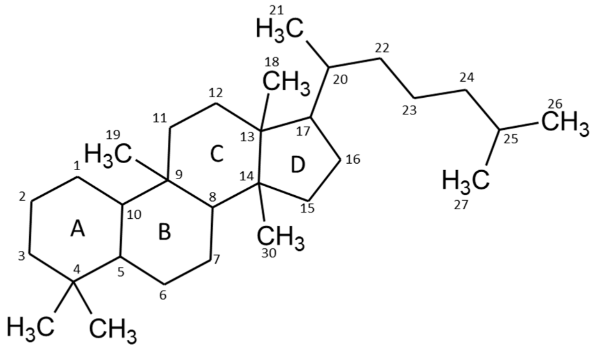

Figure S5. Illustrating the carbon numbering system for the cucurbitane skeleton.

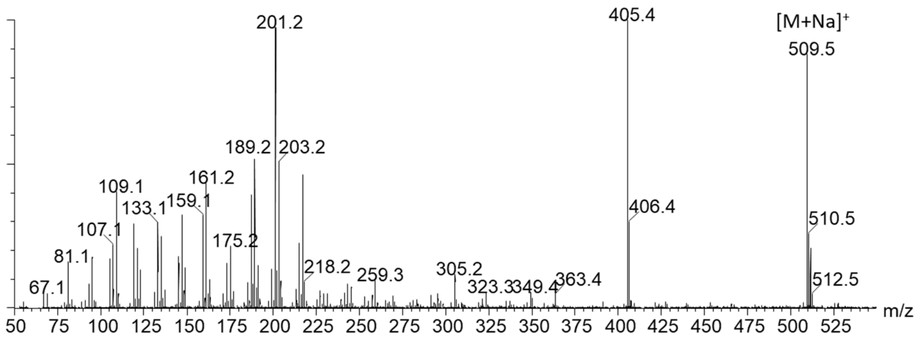

Figure S6. Fragmentation spectrum of compound **8**.

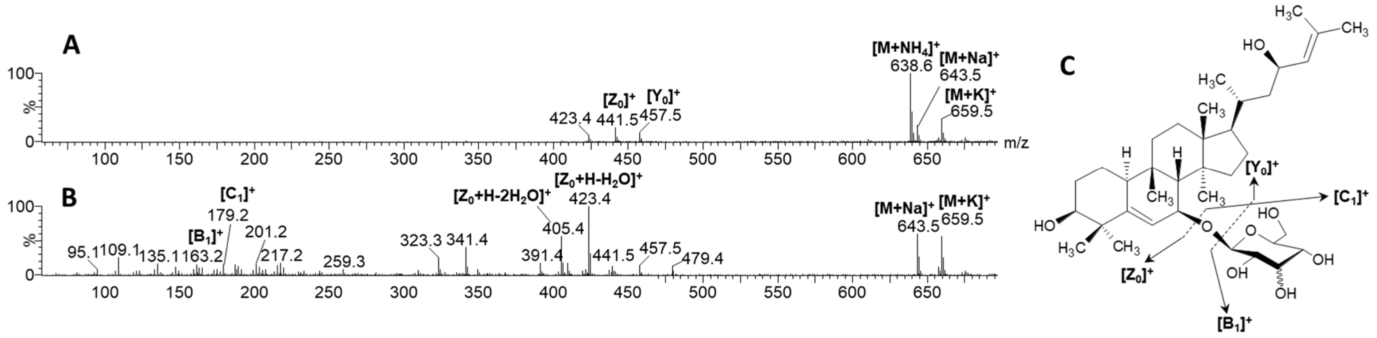

Figure S7. The low (**A**) and high (**B**) collision energy MS spectra of compound **9**, identified as a monoglycosylated cucurbitanes-type terpenoids. **C** represents the C-C bond cleavages.

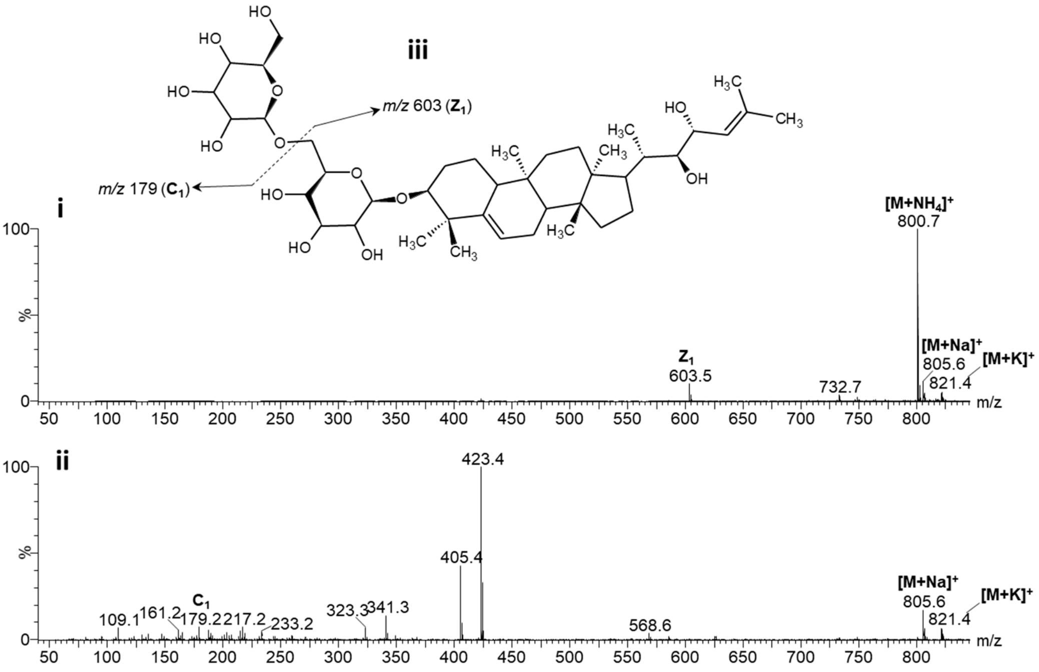

Figure S8. The low (**i**) and high (**ii**) energy spectra obtained for cucurbitane diglycosides and the proposed fragmentation pattern (**iii**).

Table S1. The Calculated $^{TW}CCS_{N2}$ Values using Poly-DL-alanine as Calibrant for Compounds **1-11.**

Compound (no)	Ionic Species Detected	Arrival Times (ms)	$^{TW}CCS_{N2}$ (Å^2)
Quercetin 3-*O*-rutinoside (Rutin) (1)	$[M+H]^+$	4.84	230.1
Kaempferol 3-*O*-rutinoside (Nicotiflorin) (2)	$[M+H]^+$	4.76	228.5
Isorhamnetin 3-*O*-rutinoside (3)	$[M+H]^+$	4.90	231.4
Balsaminol D (4)	$[M+H-H_2O]^+$	3.59	203.2
Balsaminol E, Karavilagenin E (5)	$[M+H-H_2O]^+$	4.07	216.0
Balsaminagenin C, Balsaminol F (6)	$[M+H-H_2O]^+$	3.86	210.3
Balsaminagenin A, Cucurbalsaminol A, Balsaminol A (7)	$[M+H-H_2O]^+$	4.20	219.1
		4.42	224.8
Compound 8	$[M+Na]^+$	4.69	230.9
Balsaminoside B, C (9)	$[M+NH_4]^+$	6.35	267.1

Balsaminoside A, Kuguaglycoside A (10)	$[M+NH_4]^+$	6.49	269.9
Momordicoside D (11)	$[M+NH_4]^+$	7.31	285.3

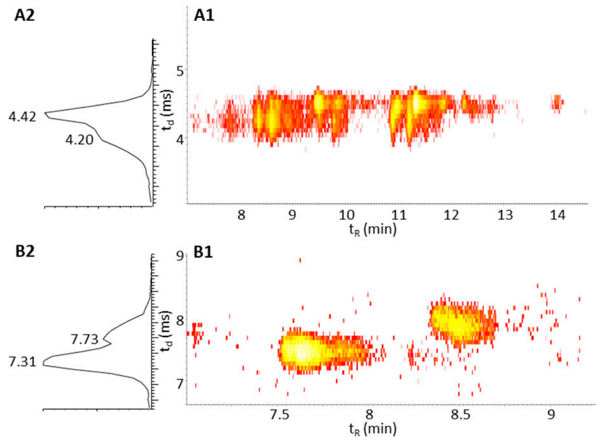

Figure S9. Extracted ion RP-LC × ion mobility contour plots for a curcurbitane aglycone (**A1**) and curcurbitane glycoside (**B1**). Panel **A2** and **B2** show the corresponding extracted ion arrival time plot.

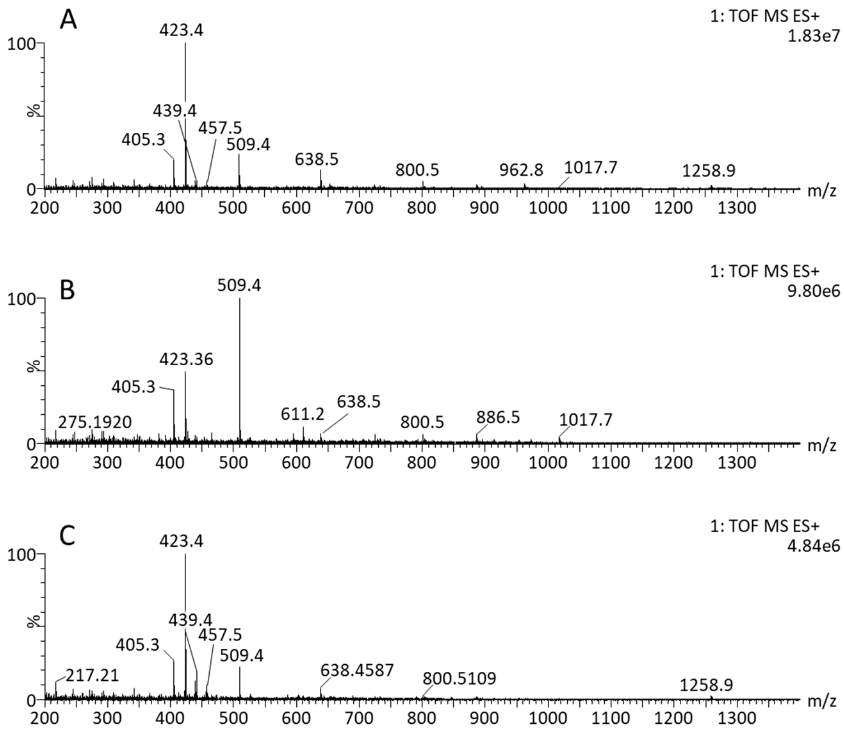

Figure S10. The QTOF MS spectra of Letsitele (**A**), Goedplaas (**B**) and Mankweng (**C**) showing the dominant ionic species in *Momordica balsamina* in the positive ionization mode.

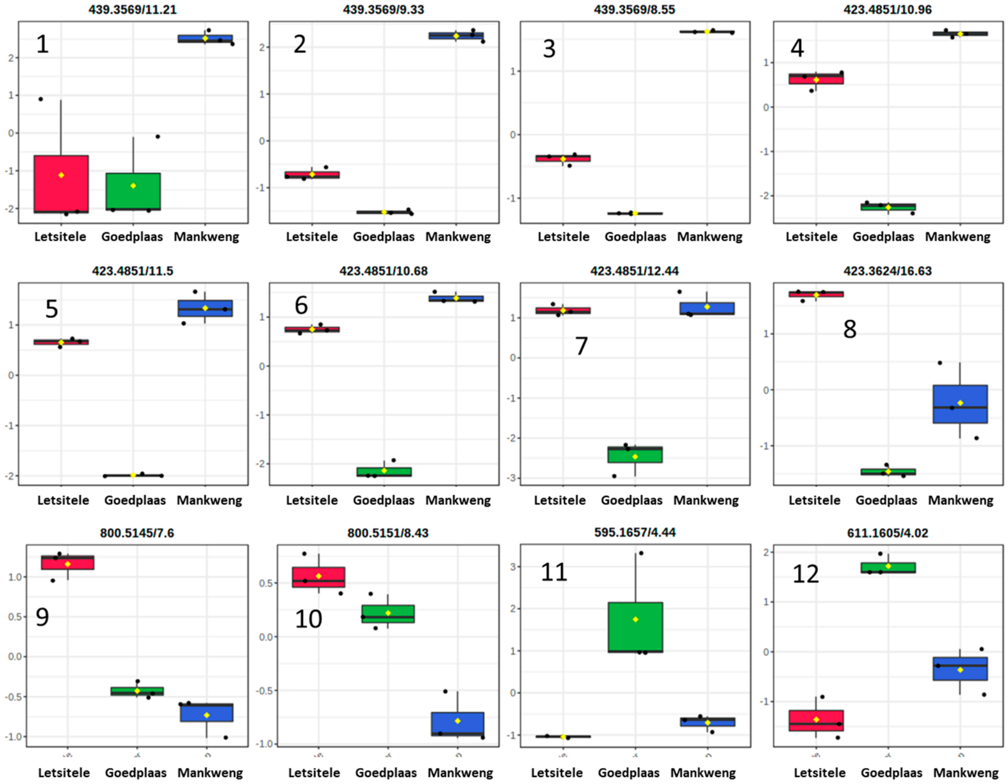

Figure S11. Boxplots illustrating the normalized concentration for selected/identified species in *M. balsamina.*

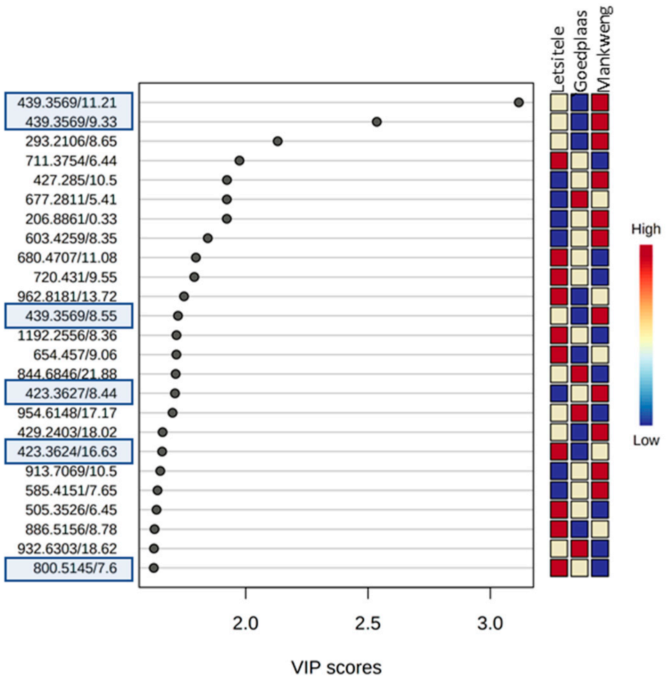

Figure S12. Variable importance in projection (VIP) plot.
